# Bacteriogenic Platinum Nanoparticles for Application in Nanomedicine

**DOI:** 10.3389/fchem.2021.624344

**Published:** 2021-03-05

**Authors:** Khalida Bloch, Karishma Pardesi, Cristina Satriano, Sougata Ghosh

**Affiliations:** ^1^ Department of Microbiology, School of Science, RK University, Rajkot, India; ^2^ Department of Microbiology, Savitribai Phule Pune University, Pune, India; ^3^ Department of Chemical Sciences, University of Catania, Catania, Italy; ^4^ Department of Chemical Engineering, Northeastern University, Boston, MA, United States

**Keywords:** platinum nanoparticles, bacteriogenic synthesis, biointerfaces, surface characterization, nanomedicine

## Abstract

Nanoscale materials have recently gained wide attention due to their potential to revolutionize many technologies and industrial sectors, including information technology, homeland security, transportation, energy, food safety, environmental science, catalysis, photonics and medicine. Among various nanoparticles, platinum nanoparticles (PtNPs) are widely used for biomedical applications, including imaging, implants, photothermal therapy and drug delivery. Indeed, PtNPs possesses intrinsic antimicrobial, antioxidant, and anticancer properties. Also, due to their remarkable catalytic activity, they are able to reduce the intracellular reactive oxygen species (ROS) levels and impair the downstream pathways leading to inflammation. Various approaches, including both physical and chemical methods, are currently employed for synthesis of PtNPs. However, the use of hazardous reaction conditions and toxic chemicals in these processes poses a potential threat to the environment and severely compromise the biocompatibility of the nanoparticles. Hereby, increasing need for exploitation of novel routes for synthesis of PtNPs has led to development of biological fabrication using microbes, specifically bacteria. Herein, we present a most comprehensive report on biogenesis of PtNPs by several bacteria like *Acinetobacter calcoaceticus, Desulfovibrio alaskensis, Escherichia coli, Shewanella algae, Plectonema boryanum*, etc. An overview of the underlying mechanisms of both enzymatic and non-enzymatic methods of synthesis is included. Moreover, this review highlights the scope of developing optimized process to control the physicochemical properties, such as the nanoparticle surface chemistry, charge, size and shape, which, in turn, may affect their nanotoxicity and response at the biointerface for nanomedicine applications.

## Introduction

Biological synthesis of nanoparticles has emerged as a promising area of nanobiotechnology which has attempted to develop environmentally benign green routes for synthesis of various nanostructures with exotic shape and sizes (Thakkar et al., 2010). Viruses, bacteria, fungi, algae and plants have been explored for their potential to synthesize gold, silver, copper, platinum, palladium, zinc and even magnetic nanoparticles (Ghosh and Webster, 2020a).

Among various metal nanoparticles platinum nanoparticles (PtNPs) are of great relevance and promising potentiality, owing to their multivariate applications in fuel cells, water gas shift reactions, electronics, petrochemical industries, organic catalysis, selective oxidation of CO, automobiles, photonics, optics, biosensors and pharmaceutics (Jeyaraj et al., 2019).

Various physical methods, such as vapour deposition, flame pyrolysis, laser ablation, sputter deposition, arc discharge, melt mixing and ball milling are used to synthesize monodispersed PtNPs. These physical methods consume enormous energy and dissipate radiations (Stepanov et al., 2014). Similarly, chemical methods for synthesis of PtNPs include the sol–gel process, plasma-enhanced chemical vapour deposition, pyrolysis, microemulsion, hydrothermal and polyol synthesis. These chemical methods involve toxic and hazardous chemicals for reduction of the metal ions to corresponding nanoparticles as well as for their stabilization (Ghosh et al., 2015). Therefore, there is need for developing clean, biocompatible, non-toxic, eco-friendly rapid and efficient methods for synthesis of PtNPs. We have reported fabrication of PtNPs and its alloy with palladium from medicinal plants like *Gloriosa superba, Barleria prionitis,* and *Dioscorea bulbifera*. Interestingly, biogenic PtNPs showed more anticancer activity compared to chemically synthesized nanoparticles (Ghosh et al., 2015; Rokade et al., 2017; Rokade et al., 2018). Fungi like *Fusarium oxyporum* and *Neurospora crassa* are reported to synthesize PtNPs either intracellularly or extracellularly with spherical, hexagonal, pentagonal, circular, squares and rectangular shapes (Ghosh et al. 2020).

Another attractive bottom up approach for synthesis of PtNPs that have attracted wide attention is bacteria mediated fabrication. Bacteria possess remarkable ability to reduce metal ions to corresponding nanoparticles which are highly stable with notable therapeutic properties. Hence, a wide number of bacterial species like *Acetobacter xylinum, Acinetobacter calcoaceticus, Desulfovibrio alaskensis, Escherichia coli, Shewanella algae, Plectonema boryanum*, and so forth are reported for synthesis of PtNPs with different shapes, sizes and biological activity (Gahlawat and Choudhury, 2019; Ghosh and Webster, 2020b). The synthesis of nanoparticles achieved by biogenic enzymatic process are excellent as compared to other methods. The NPs produced by this method showed greater catalytic potential. Microorganism adapt different environmental conditions and they produces NPs in order to generate energy and detoxify the toxic molecules to survive in adverse environments.

In this review an elaborate account of advances in the area of bacteriogenic synthesis of PtNPs is presented. Further, we have presented the mechanism of synthesis and the applications of bacteriogenic PtNPs. Finally, the limitations of microbial synthesis and the scope to overcome them are also discussed.

### Synthesis, Advantages and Disadvantages

**FIGURE 1:**
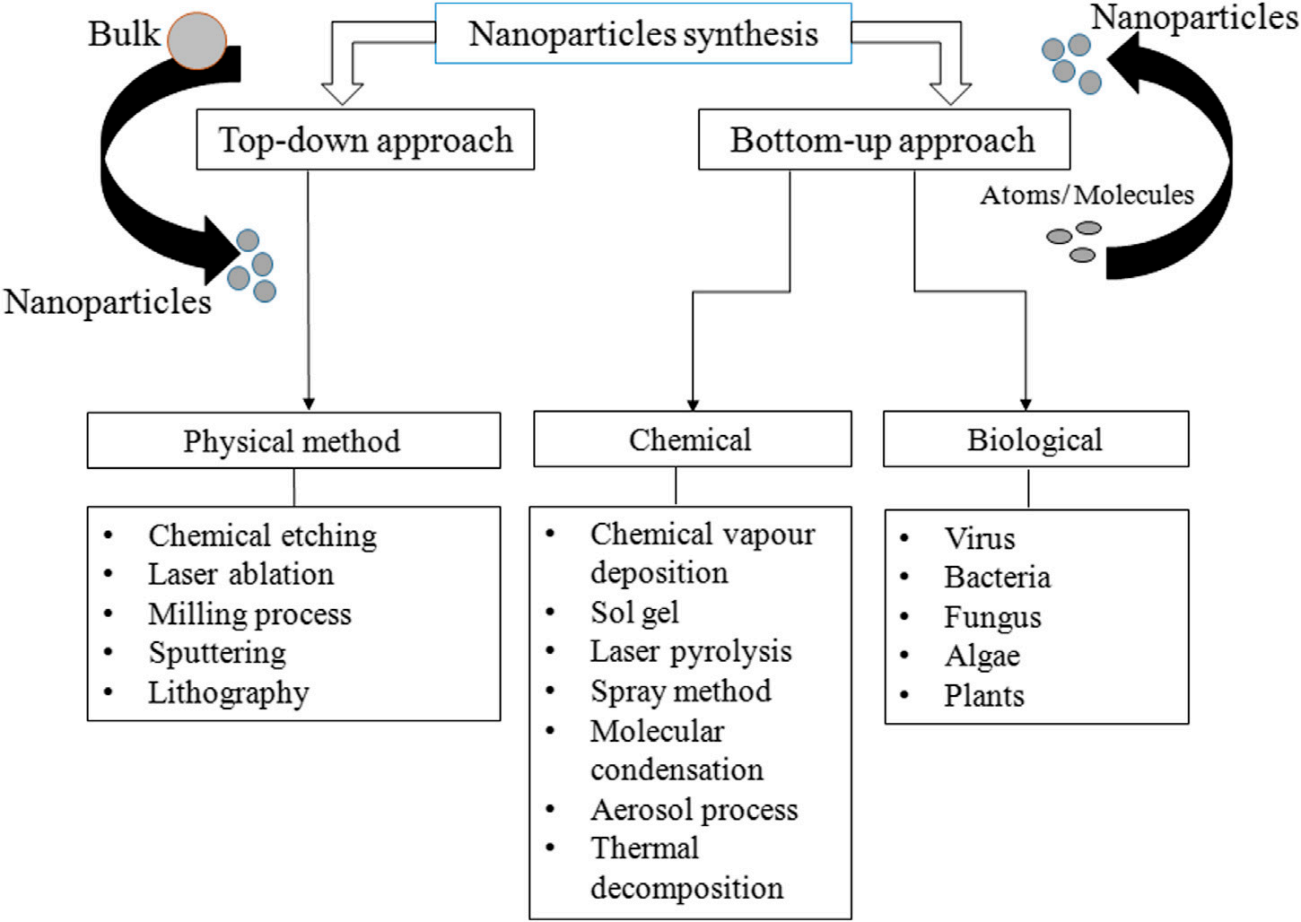
Various methods for synthesis of PtNPs.

Various methods are used for the synthesis of PtNPs, which are broadly divided into two major categories: top-down and bottom-up approaches. In top-down approaches, metal in bulk amount is used for the synthesis of nanoparticles via mechanical breakdown (Dhand et al., 2015). The major advantage is uniform size and shape of resulting PtNPs. Several physical methods are used for the synthesis of PtNPs such as lithography, thermal decomposition, chemical etching and sputtering (Mafune and Kondow, 2004; Park et al., 2010; Cueto et al., 2011). In bottom-up approach atoms and molecules assembles to generate range of NPs (Nichols et al., 2006). Sol-gel processing, laser pyrolysis, chemical vapour deposition and plasma spraying synthesis are included in bottom up approach. Synthesis methods are divided into three groups- physical, chemical and biological as depicted in Figure 1. The physical method involves high mechanical pressure, energy, and include evaporation and condensation to generate PtNPs. Table 1 shows the advantages of physical method that include no use of toxic chemicals, purity, uniform size and shape whereas its disadvantage includes high cost, exposure to radiation, high temperature and less productivity. It also changes the physicochemical property of nanoparticles.

**TABLE 1:** Advantages and disadvantages of different methods used for synthesis of PtNPs.

Methods	Advantage	Disadvantage
Physical method	High speed, no use of toxic chemicals, purity, uniform size and shape.	Productivity, high cost, exposure to radiation, require high energy, temperature and pressure, large amount of waste generation, high dilution, difficult size and shape tunability, lower stability, altered surface chemistry and physicochemical properties of nanoparticles.
Chemical method	Cost effective, high versatility in surface chemistry, easy functionalization, high yield, size controllability, thermal stability, reduced dispersity.	Low purity, use of toxic chemicals and organic solvents, hazardous to human beings and environment.
Bacteriogenic synthesis	Simple, facile ecofriendly, nontoxic and biocompatible.	Hard to control size, shape, crystal growth. Stability and aggregation, possible presence of endotoxin, time consuming purification processes.

On the other hand chemical synthesis of PtNPs involves interaction of atoms and smaller molecules where the precursor metal ions are converted to corresponding nanoparticles. Water soluble precursors are used to enhance the reduction of metal monomers. Various synthesis techniques include sol-gel process, microemulsion, hydrothermal, polyol synthesis and pyrolysis (Bonnemann and Richards, 2001; Liu et al., 2002; Kyung Tae et al., 2013). The chemical synthesis is cost effective, highly versatile, high yielding, controllable, thermally stable and less dispersed but it also includes low purity, use of toxic chemicals and hence hazardous to human beings and environment.

Biological synthesis has recently gained much attention for the synthesis of PtNPs. The advantage of green synthesis is that they are simple, facile, ecofriendly, non toxic and biocompatible (Naseer et al. 2020). PtNPs with definite shape and size can be produced by changing the concentration of reducing agent, temperature and pH. The synthesis of PtNPs is limited as compared to synthesis of silver, gold and other metal NPs. Biological systems such as bacteria, fungi and plants are considered the potentially environment friendly nanofactories. Green synthesis are alternatives to physical and chemical methods as they are non toxic, cost effective, gives rapid synthesis, ecofriendly, monodispersed, low in waste production and also give large scale production (Jeyaraj et al., 2019).

## Bacteriogenic Platinum Nanoparticles

Several bacteria have the ability to biosorb metal ions on their surface and eventually reduce them to the corresponding nanoparticles by various mechanisms that may include reductases, cytrochromes and metallothioneins (Patel and Ponnuchamy, 2019; Gautam et al. 2020). Bacterial cellulose, rhizospheric bacteria, anaerobic sulphate reducing bacteria and even photoautotrophic cyanobacteria can synthesize PtNPs with exotic shape and size as presented in Table 2. In the following section bacteria with potential to synthesize PtNPs are discussed in details.

**TABLE 2:** Bacteria mediated synthesis of PtNPs.

Bacteria	Size	Shape	Localization	References
*Acetobacter xylinum*	6.3–9.3 nm	Granular	Extracellular and intracellular	Aritonang et al. (2014)
*Acinetobacter calcoaceticus* PUCM 1011	2–3.5 nm	Cuboidal	Intracellular	Gaidhani et al. (2014)
*Desulfovibrio alaskensis* G20	-	-	Extracellular	Capeness et al. (2015)
*Desulfovibrio vulgaris* Hildenborough (DSM 644)	-	-	Extracellular	Martins et al. (2016)
*Escherichia coli* MC4100	2.3 ± 0.7 nm and 4.5 ± 0.7 nm	Spherical	-	Attard et al. (2012)
*Plectonema boryanum* UTEX 485	30 nm–0.3 µm	Spherical	Intracellular and extracellular	Lengke et al. (2006)
*Calothrix pulvinata* ALCP 745A	3.2 ± 0.3	Well-shaped	Intracellular	Brayner et al. (2007)
*Anabaena flos-aquae* ALCP B24	-	-	Intracellular	Brayner et al. (2007)
*Leptolyngbya foveolarum* ALCP 671B	-	-	Intracellular	Brayner et al. (2007)
*Pseudomonas aeruginosa* SM1	450 nm	Circular disk like	Extracellular	Srivastava and Constanti, (2012)
*Shewanella algae*	5 nm	Discrete	Periplasmic	Konishi et al. (2007)
*Shewanella oneidensis* MR-1	3–40	Spherical	Intracellular and extracellular	Xu et al. (2019)
	61.03	Snowflake shape	extracellular	Tuo et al. (2016)
*Streptomyces* sp*.*	20–50 nm	Spherical	Extracellular	Baskaran et al. (2017)
*Sulphate reducing bacteria*	200–1000 nm	Irregular, rectangle and square	Cell free soluble extract	Riddin et al. (2010)
*Jeotgalicoccus coquinae* ZC15	5.74	Spherical	Extracellular	Eramabadi et al. (2020)
*Kocuria rosea* MN23	5.85	Spherical	Extracellular	Eramabadi et al. (2020)
*Pseudomonas kunmingensis* ADR19	3.95	Spherical	Extracellular	Eramabadi et al. (2020)
*Pseudomonas putida* KT2440	8.06	Spherical	Extracellular	Eramabadi et al. (2020)
*Psychrobacter faecalis* FZC6	2.49	Spherical	Extracellular	Eramabadi et al. (2020)
*Sporosarcina psychrophila* KC19	4.24	Spherical	Extracellular	Eramabadi et al. (2020)
*Vibrio fischeri* NRRL B-11177	3.84	Spherical	Extracellular	Eramabadi et al. (2020)

## *Acetobacter xylinum*

**FIGURE 2:**
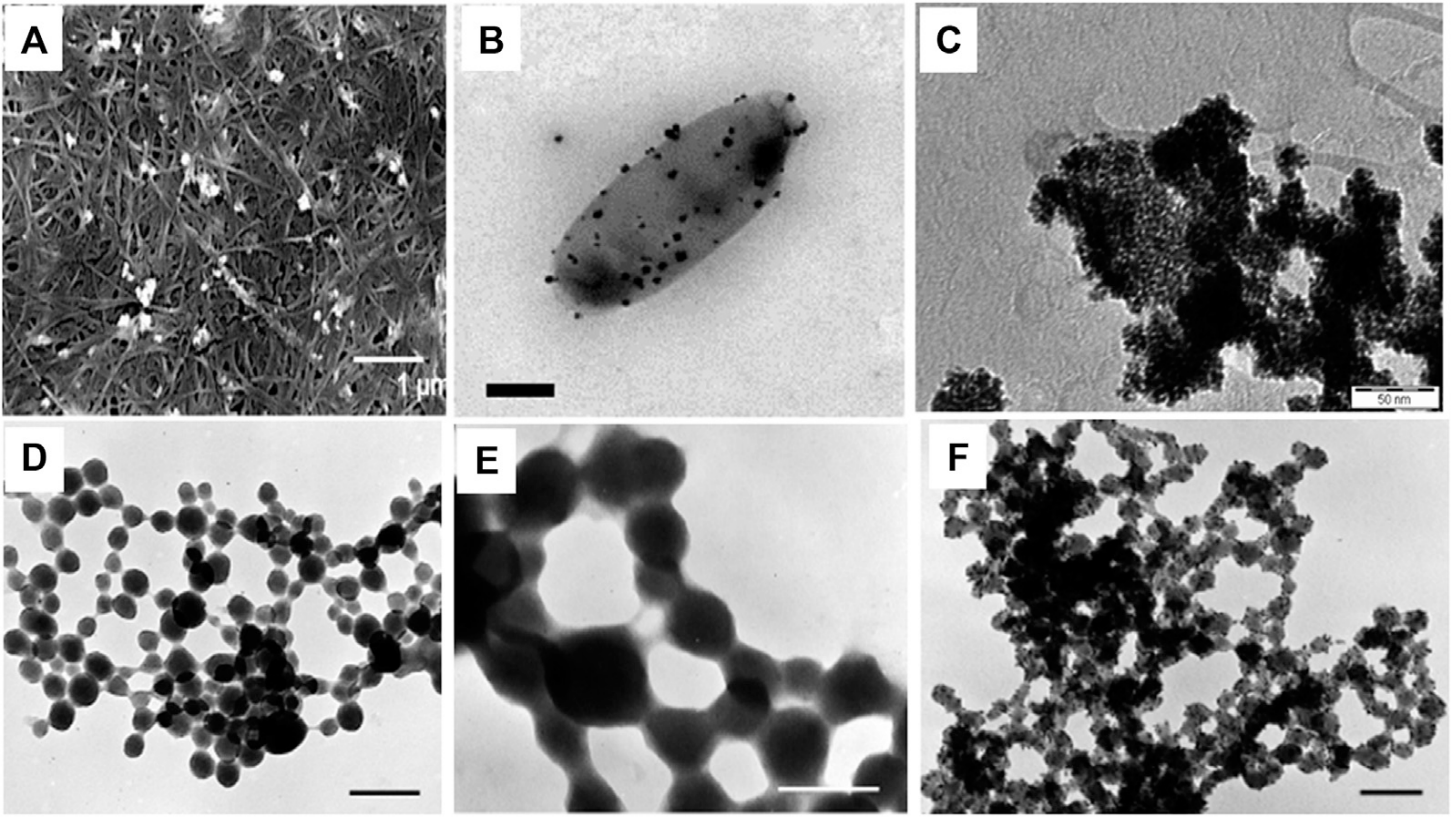
Morphologies of bacteriogenic PtNPs. **(A)** SEM images of Pt-BCcm membrane obtained from K_2_PtCl_4_ solution at 10mM where inset scale bar represents 1 µm (Aritonang et al., 2014); **(B)** Representative electron micrographs of PtNPs produced by *D* ***.*** *alaskensis* G20 which are exported and found on the surface of the cell where inset scale bar represents 200 nm (Capeness et al., 2015); **(C)** TEM image of recovered bioPt NPs from 1% Pt EcMC4100 after NaOH, washing, and centrifugation treatment where inset scale bar represents 50 nm (Attard et al., 2012); TEM micrographs of whole mounts of partially recrystallized spherical PtNPs in cyanobacteria-PtCl_4_° systems at **(D)** at 60°C and 14 days where inset scale bar represents 0.25 µm; **(E)** at 80°C and 21 days where inset scale bar represents 0.1 µm; **(F)** 100°C days and 28 days where inset scale bar represents 0.1 µm (Lengke et al., 2006).

Synthesis of platinum nanoparticles (PtNPs) was achieved using bacterial cellulose (BC) matrix of *A. xylinum*. Dry BC membrane was used for the synthesis of PtNPs where initially BC membrane was allowed to soak with different concentration of aqueous K_2_PtCl_4_ solution for 2 h followed by sonication at room temperature. Membrane was then washed with deionized water. Hydrogen gas was applied at 0.5 psi and stirred at 500 rpm for 1 h at room temperature to reduced Pt(II). Pt-BC gel was then dried and dry membrane composite was produced with thickness of 0.5–0.7 mm. The colour change of BC membrane from transparent white to black indicated deposition of Pt on the fibres of BC. White granules were found to be embedded on BC membrane which was revealed by SEM images. Figure 2A shows that the PtNPs varied in particle size ranging from 6.3–9.3 nm with varying salt concentration (Aritonang et al., 2014).

### *Acinetobacter calcoaceticus* PUCM 1011

*A. calcoaceticus* PUCM 1011, isolated from rhizosphere of *Pennisetum glaucum* was used for the synthesis of PtNPs. The bacteria were grown in Luria Bertani (LB) broth to obtain the cell pellet which was then suspended in sterile distilled water to get cell density 1 McFarland unit. Aqueous solution of hexachloroplatinic acid (H_2_PtCl_6_) was mixed with cell pellet to get a final concentration of 1 mM. The resulting reaction mixture was allowed to incubate for 168 h at 30°C. The reaction mixture was held at different salt concentration (1–10 mM), pH (5–9) and temperature (20–50°C). Sharp absorption peak at 300 nm was displayed in the UV-Vis spectra of the bacteriogenic PtNPs. Intracellular cuboidal PtNPs of size ranging from 2–3.5 nm were synthesized. It was found that 1.07 x 10^–3^ mol/L of PtNPs were formed within 72 h at 30°C with 1 mM salt concentration. The PtNPs synthesized from purified bacterial cell proteins were in size ranging from 1–4 nm and also few were in picometer range (Gaidhani et al., 2014).

### *Desulfovibrio alaskensis* G20

Anaerobic sulfate reducing bacteria *D. alaskensis* G20 was used for the synthesis of PtNPs. The organism was grown on Postgate Media C (PGMC) where lactate was used as carbon source and was incubated in anaerobic hood fed with 10% CO_2_ and H_2_ at 30°C. The cell pellet was obtained by centrifuging the cells and was suspended in 10 mM 3-(N-morpholino)-propanesulfonic acid (MOPS) buffer with pH 7. The 2 mM PtCl_4_ salt was added to the cell suspension and was allowed to incubate for 2 h. Figure 2B depicts that the PtNPs were produced extracellularly and were adhered to the cell surface (Capeness et al., 2015).

### *Desulfovibrio vulgaris* Hildenborough (DSM 644)

PtNPs were also biologically synthesized using *D. vulgaris* Hildenborough (DSM 644). Modified Postgate media C (pH 7.2) (0.5 g/L KH_2_PO_4_, 1 g/L NH_4_Cl, 2.5 g/L Na_2_SO_4_, 0.06 g/L CaCl_2_.2H_2_O, 0.06 g/L MgSO_4_.7H_2_O, 1 g/L yeast extract, 0.0071 g/L FeSO_4_.7H_2_O, 0.3 g/L sodium citrate tribasic dihydrate, 0.1 g/L ascorbic acid, 0.1 g/L sodium thioglycolate, 4.5 g/L sodium lactate and 0.3 g/L resazurin) was used for the growth of organism. The cells were harvested by centrifugation and were washed with anaerobic MOPS/NaOH buffer (20 mM, pH 6.8). The cell suspension was added to metal salt solution (100 mg/L, pH 2.5) and the reaction mixture was kept under anaerobic condition. After 1 h of incubation under an atmosphere of hydrogen, the reaction mixture was kept overnight at 37°C under 0.8 bar pressure. Bioreduced PtNPs were deposited on the cell surface which confirmed extracellular mode of synthesis (Martins et al., 2016).

### *Escherichia coli* MC4100

In another study *E. coli* MC4100 was grown aerobically on nutrient agar plate at 30°C while for catalyst preparation the bacteria were grown anaerobically in nutrient broth (NB) at 37 °C (pH 7.3). Bacterial culture was harvested under oxygen-free nitrogen (OFN) by centrifugation and was treated with degassed 20 mM Na-MOPS (3-(N-morpholino) propanesulfonic acid) buffer. Cell culture was then transferred anaerobically into serum bottle and mixed with degassed 2 mM of Na_2_PtCl_4_ to achieve the final ratio of 1:99 and 1:5 (weight of Pt: dry weight of cells). The reaction mixture was incubated for 30 min at 30°C to allow biosorption of Pt(II) on the cell surface followed by passage of hydrogen gas for 10 min at 20°C at pH 2.3 that led to reduction of Pt(II) to Pt(0). The bioreduced PtNPs were harvested by centrifugation and were kept for 6 weeks with 5.5 M NaOH for the removal of organic material coating. The PtNPs were then deposited on gold electrode for the electrochemical measurements. Figure 2C illustrates that the spherical PtNPs (with 1% by mass metal loading) were 2.3 ± 0.7 nm in diameter while spherical PtNPs generated with 20% metal loading exhibited an average size of 4.5 ± 0.7 nm (Attard et al., 2012).

### *Plectonema boryanum* UTEX 485

PtNPs were synthesized employing cyanobacteria, *P. boryanum* UTEX 485 that was grown in batch culture using BG-11 medium buffered with 10 mM HEPES at 29°C in presence of CO_2_. The cyanobacteria were allowed to grow for 6–8 weeks and cells were harvested by centrifugation and then 5 ml of PtCl_4_ (∼500 mg/L) was added to 5 ml cyanobacterial culture. The reaction mixture was held at different temperature (25, 60, 80 and 100°C) for 28 days. The reaction was also allowed to incubate at 180°C for 1 day in order to check the effect of temperature on morphology. SEM analysis revealed that the shape of the particle (incubated at 25°C) was found to be spherical with size ranging from 30 nm to 0.3 µm. TEM-EDS showed the presence of platinum with sulfur, iron and chloride. At 60 °C and 80°C, particles were spherical with size 30 nm to 0.2 µm which formed long beadlike chains (Figures 2D,E). At 100°C, very fine grained PtNPs were observed as seen in Figure 2F. However, at 180°C PtNPs formed a branched pattern exhibiting a dendritic morphology. X-ray Photoelectron Spectroscopy (XPS) was carried out to investigate oxidation state of platinum and sulfur (Lengke et al., 2006).

Among several other cyanobacteria *Anabaena*, *Calothrix*, and *Leptolyngbya* were also used for the synthesis of PtNPs. *Calothrix pulvinata* ALCP 745A, *Anabaena flosaquae* ALCP B24, *Leptolyngbya foveolarum* ALCP 671B were cultivated in sterile Bold’s Basal medium for different time periods like one month, 20 days and 6 days. *In-vitro* enzymatic reduction assay was carried out to confirm that nitrogenase is responsible for the reduction of PtNPs. Bold’s Basal medium with nitrogenase enzyme as reducing agent was used along with *Calothrix* released polysaccharides (RPS) in order to simulate *in vivo* conditions. The size of PtNPs synthesized from *Calothrix* was found to be 3.2 ± 0.3 nm. The binding energy after peak decomposition were found to be 71.0, 73.4 and 75.8 eV for *Calothrix* and 71.5, 73.7 and 75.8 eV for *Anabaena* (Brayner et al., 2007).

### *Pseudomonas aeruginosa* SM1

*P. aeruginosa* SM1 was used for the extracellular synthesis of PtNPs where the organism was initially cultured in nutrient broth (10 g/L peptone, 10 g/L meat extract and 0.5 g/L NaCl) and was incubated at 27°C under shaking condition (120 rpm). Then bacterial cell pellet (0.2 g) was added to 0.001 M of ammonium hexachloroplatinate (IV) ((NH_4_)_2_PtCl_6_) solution and the reaction mixture was kept at 25°C. Morphological analysis using TEM showed that the PtNPs were circular disk like with a size of 450 nm. The primary and secondary amines in the bacterial metabolites were reported to be responsible for the synthesis of extracellular PtNPs (Srivastava and Constanti, 2012).

### *Shewanella algae*

*S. algae* ATCC 51181 was used for the biological synthesis of PtNPs where the bacterial strain was anaerobically cultured in ATCC medium 2 containing sodium lactate in presence of N_2_-CO_2_ (80:20, v/v). Cells were recovered by centrifugation and suspended in bicarbonate buffer. Cell suspension was added into 10 ml of aqueous H_2_PtCl_2_ solution to achieve a final cell concentration of 1 × 10^9^ cells/ml and the reaction mixture was incubated at 25°C. The colour change from pale yellow to black within 60 min indicated formation of discrete PtNPs which were 5 nm in size and were located in periplasmic space between inner and outer membrane of the bacterial cell (Konishi et al., 2007).

### *S. oneidensis* MR-1

Biogenic synthesis of PtNPs was checked using *S. oneidensis* MR-1 (ATCC 700550) in absence and presence of quinone. Mineral salt medium was inoculated with cell in which formate was used as electron donor and H_2_PtCl_6_ (H_2_O)_6_ salt as precursor for the synthesis of PtNPs. Reaction took place in dark anaerobically at 30°C for 24 h that led to 100% reduction of metal salt to PtNPs that was indicated by the appearance of black colour. Small clusters of snowflake shaped PtNPs were observed on the surface of the bacterial cell with size of 61.03 nm. Further, addition of Anthraquinone-2,6- disulfonate (AQDS) enhanced the reduction process resulting in smaller and larger PtNPs with 2.45 nm and 120 nm sizes, respectively (Tuo et al., 2016).

Green synthesis of Pd-Pt alloys NPs was reported using *S. oneidensis* MR-1 in presence of mixed solution of 100 mg/L of Pd (II) and Pt (IV). Sodium formate was added and the reaction mixture was allowed to incubate for 3 h at 30°C resulting in synthesis of Pd-Pt NPs both intracellularly and extracellularly which were mostly spherical with 3–40 nm size. Also spherical extracellular nanoparticles with size of 12–40 nm were found to be located onto the surface of the cell with average size of 26.0 nm. Hence the extracellular particles were larger in size than the intracellular. The surface enzymes, proteins, reducing components of bacteria aided in reduction of metal ion. Amines, carbonyl and hydroxyl functional group of protein and polysaccharides might have played a critical role as reducing and stabilizing agent during biosynthesis of Pd-Pt alloys NPs (Xu et al., 2019).

### *Streptomyces* sp.

PtNPs were synthesized from *Streptomyces sp.* isolated from Kodiyakarai marine segment of Tamil Nadu, India. The biomass of *Streptomyces* sp. was recovered by centrifuging at 10,000 rpm followed by filtration through polyethersulphone filter to produce cell free supernatant (CFS). Different concentration of CFS was mixed with aqueous potassium hexacloroplatinate (IV) (K_2_PtCl_6_) solution (1 mM) and was incubated for 24 h at 30°C. UV-Vis spectra showed absorption peak at 262 nm indicating synthesis of PtNPs which were face centered cubic (FCC) of crystalline nature with size of 24.1 nm. Atomic force microscopy (AFM) and TEM analyses confirmed that the size of the bacteriogenic PtNPs were spherical with 20–50 nm size (Baskaran et al., 2017).

### Sulphate Reducing Bacteria (SRB)

SRB were isolated from sewage sludge obtained from Environmental Biotechnological Research Unit (EBRU) Bio-sure Process (Grahamstown, South Africa) was used for the synthesis of PtNPs. The organism was cultured under anaerobic conditions in dark at room temperature by exposing to N_2_ (99.9%) and H_2_ (99%) for 15 min and 5 min, respectively (Riddin et al., 2009). The SRB cells were harvested and pellet was washed with double distilled water to remove sulfide or sulfate. The cell-free soluble extract (CSE) was incubated on ice for 10 min and 250 µg/ml protein from CSE was mixed with H_2_PtCl_6_ such that the ratio of salt solution to protein varied from 0.7:1 to 4:1. The pH was adjusted to 9.0 and the reaction mixture was incubated in dark at 65°C. The gradual color change from light yellow to brown indicated formation of PtNPs. Lower percentage of soluble protein resulted in slow reduction of platinum salt. TEM analysis revealed that 0.7:1 sample showed irregular, electron dense particles with 200–1000 nm size while sample with 1:1 ratio had particle size 100–500 nm with rectangles and squares shapes. Particle size of PtNPs were in range from 200–800 nm with irregular shape when the reactant ratio was 1.5:1 (Riddin et al., 2010).

### Microbial Cell Lysate Supernatant (CLS)

In another study, synthesis of PtNPs were carried out using microbial cell lysate supernatant derived from various Gram-positive and Gram-negative bacteria such as *Pseudomonas kunmingensis* ADR19, *Psychrobacter faecalis* FZC6, *Vibrio fischeri* NRRL B-11177, *Jeotgalicoccus coquinae* ZC15, *Sporosarcina psychrophila* KC19, *Kocuria rosea* MN23 and *Pseudomonas putida* KT2440. The bacterial strains were cultivated at 30°C for 48 h at 200 rpm in Luria-Burtani broth (LB,tryptone 10 g/L, sodium chloride 10 g/L, yeast extract 5 g/L) medium and 1g of cell pellet was suspended in SDW and further sonicated for 5 min thrice at interval of 30 s. Cell debris were removed by centrifugation and the supernatant was filtered to obtain cell lysate supernatant (CLS). After adjusting the pH of the resulting CLS, H_2_PtCl_6_.7H_2_O was mixed and incubated for 1 h at 90°C which led to the synthesis of PtNPs with average particle size of 3.95, 2.49, 3.84, 5.74, 4.24, 5.85 nm for *P. kunmingensis* ADR19, *P. faecalis* FZC6, *V. fischeri* NRRL B-11177, *J. coquina* ZC15, *S. psychrophila* KC19, *K. rosea* MN23, respectively. The size of PtNPs synthesized from genetically engineered bacterium *P. putida* KT2440 was found to be 8.06 nm (Eramabadi et al., 2020).

## Mechanism Behind PtNPs Synthesis

**FIGURE 3:**
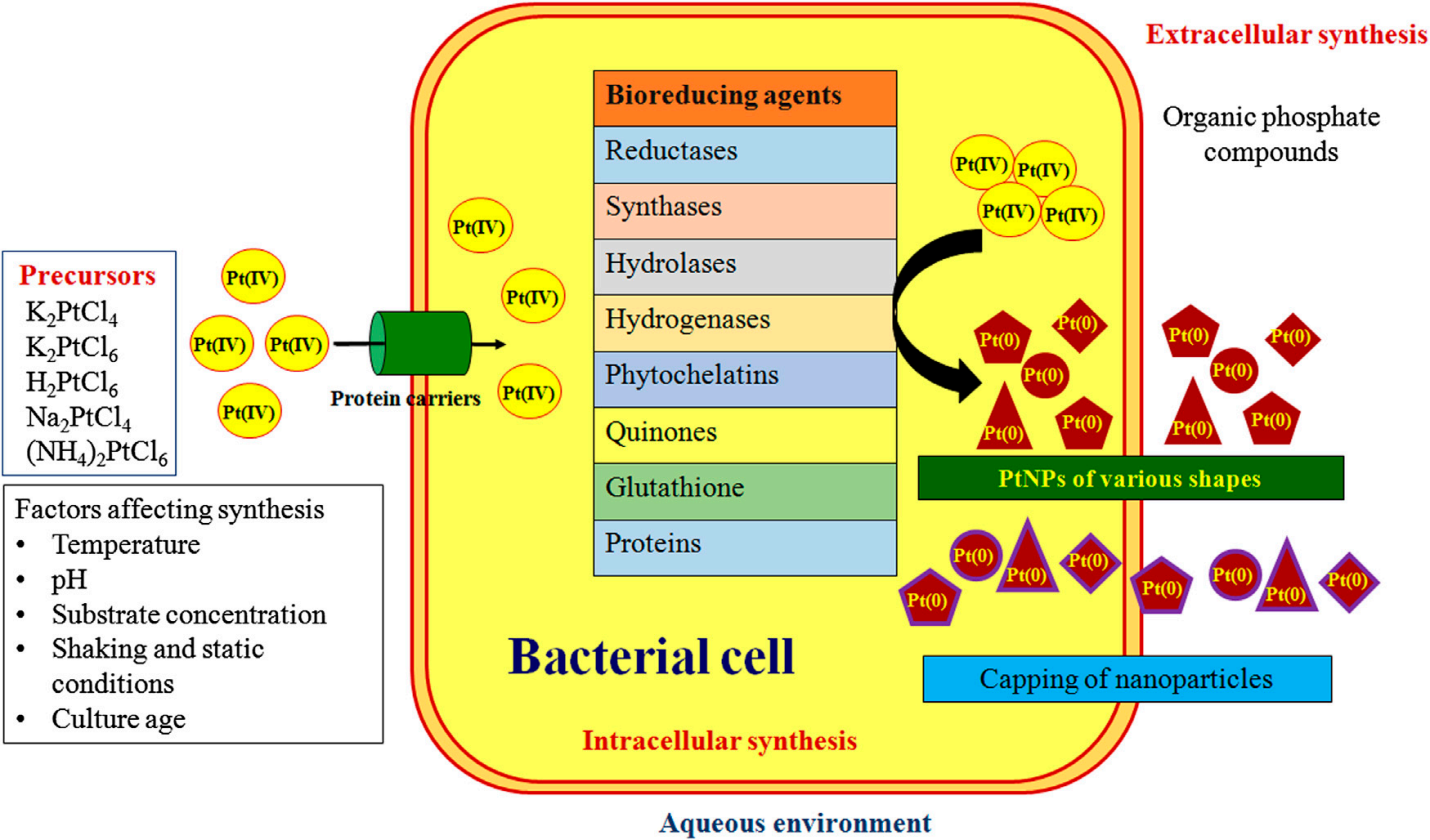
Extracellular and intracellular bacterial synthesis of nanoparticles

Numerous mechanisms were reported for the bacterial synthesis of PtNPs which are depicted in Figure 3. Hydrogen gas was used as reducing agent during synthesis of PtNPs using bacterial cellulose (BC) from *Acetobacter xylinum.* The K_2_PtCl_4_ in solution undergoes solvolysis resulting in PtCl_2_(H_2_O_2_)_2_ and thus hydrogen bonding was found between oxygen atom of BC and hydrogen atom of PtCl_2_(H_2_O_2_)_2_ and vice versa. PtNPs were reduced on surface and inner side of BC membrane (Aritonang et al., 2014). *A. calcoaceticus* PUCM 1011 showed four protein bands with molecular mass 97, 66, 43 and 29 kDa, respectively that were responsible for synthesis of PtNPs. Hence, a multistep process was proposed to be involved in reduction of Pt(IV) to PtNPs intracellularly, that were later released due to rupture of the cell membrane leading to cell death. Bacterial proteins were also responsible for stabilization of the biogenic nanoparticles (Gaidhani et al., 2014). Synthesis of PtNPs by anaerobic sulfate reducing bacteria*, D. alaskensis* G20 was attributed to cytochromes and/or hydrogenases enzymes. After the formation they are exported to the outer surface of the cell hence forming extracellular PtNPs (Capness et al., 2015).

Another bacteria, *D. vulgaris* Hildenborough (DSM 644) encodes seven putative Hases enzymes out of which four are located in periplasm and three in cytoplasm. These enzymes might play a critical role in synthesis of PtNPs. The extracellular PtNPs strongly rationalize the fact that the periplasmic Hases were involved in reduction of metal ion into nanoparticle. Furthermore, bacterial surface may act as best nucleation site for metals adsorption hence supporting the deposition of NPs on cellular surface (Martins et al., 2016). *E. coli* MC4100 possesses various hydrogenases within cell that helps in catalyzing the hydrogen into proton and electrons that may facilitate the biogenic synthesis of PtNPs (Attard et al., 2012).

Cyanobacteria such as *P. boryanum* UTEX 485 were also reported to synthesize PtNPs efficiently both intracellularly and extracellularly. Polysaccharides in the cell envelope with high uronic acid content provide carboxyl groups that facilitate binding of metal to the bacterial cell surface. Further, metallothioneins can bind to form metal-thiolate interactions due to sulphur rich amino acids in the polypeptides. Formation of dispersed PtNPs in *P. boryanum* might be attributed due to the entry of platinum into the cells as complexed PtCl4° [Pt(IV)] that was successively reduced to Pt(II) and then to Pt(0) by cyanobacteria. Although, contact with the platinum(IV)-chloride reagent or the low pH and the high temperatures (60–180°C) immediately resulted in the death of the live biomass, the released metabolites might have played an essential role in the further synthesis and precipitation of PtNPs. Also, association of sulfur with bioreduced PtNPs indicated the involvement of organic sulphur and possibly organic phosphorus in the reduction and complexation of PtCl_4_° (Lengke et al., 2006).

In other cyanobacteria reduction of metal salt to PtNPs occurred inside the heterocyst and vegetative cells followed by partial release of PtNPs into the medium. A reducing enzyme, nitrogenase is present in the cells which could be responsible for the reduction of platinum salt into PtNPs. Cyanobacteria possess two class of reducing enzymes, nitrogenase and hydrogenase. Nitrogenase is present in heterocysts of *Calothrix* and *Anabaena* which is responsible for the fixation of nitrogen from atmosphere. *Anabaena variabilis* produces two different nitrogenases which can function under either anaerobic or aerobic conditions present in heterocyst while other can function only in aerobic condition in heterocyst and vegetative cells. Hydrogenase reduces hydrogen ion into molecular hydrogen which may be involved in the reaction. Hence nitrogenase and hydrogenase can play a critical role for synthesis of PtNPs in cyanobacteria. *Leptolyngbya* does not possess heterocyst and fixes nitrogen only in dark hence gives less reduction efficiency. As the enzyme is found to be located in fibrous layer inside the heterocyst and thylakoids of vegetative cells, no PtNPs were observed in intra-thylakoid and intra-fibrous layer. Hence membrane controlled biomineralization might play a crucial role in controlled growth of PtNPs (Brayner et al., 2007).

*S. algae* synthesized PtNPs in periplasm and not in aqueous solution. Hence enzymes responsible for the reduction of PtCl_6_^2-^ might be localized in the periplasm. The synthesis of PtNPs using *S. algae* was thought to be a two-step process where initially the PtCl_6_^2-^ ion uptake from aqueous solution into periplasmic space occurred which was followed by enzymatic reduction of ions into PtNPs using lactate as electron donor within 60 min at pH 7 and 25°C (Konishi et al., 2007).

Riddin et al. (2010) reported involvement of two different hydrogenase enzymes for synthesis of PtNPs. Initially, the platinum(IV) was reduced to platinum(II) by a two-electron bioreduction using an oxygen tolerant/protected novel cytoplasmic hydrogenase. Second with the help of another two-electron bioreduction involving an oxygen sensitive periplasmic hydrogenase, the platinum(II) ion was reduced to platinum(0) nanoparticles. Alternatively, the extracellular synthesis of PtNPs by *Streptomyces* sp. was attributed to the bacterial chloride reductase enzyme which is generally involved in nitric acid cycle and plays an important role in the reduction of chloride to chlorine. Nicotinamide adenine dinucleotide dependent chloride reductase enzyme might play a vital role in synthesis of PtNPs where the Pt ions were probably reduced via an electron shuttle enzymatic metal reduction process (Baskaran et al., 2017).

The proper proposed mechanism for the synthesis of nanoparticles by microorganism has not been conceived yet as different organism react differently during the synthesis of nanoparticles. Microorganism produces nanoparticles intracellularly and extracellularly. In intracellular synthesis, cell wall plays a crucial role in which electrostatic interaction of positive charge metal ion with negative charge cell wall takes place. The ion transport system transfer the ions inside the cell and the enzymes present inside the cell reduces the metal ions into nanoparticles. Many enzymes such as reductases, synthases, hydrolases and hydrogenases play role in synthesis and stabilization process. NADH and NADPH are commonly employed. In extracellular synthesis, microbial enzymes secreted in medium subsequently reduces metal ions into their respective nanoparticles and also they act as capping agent (Chaudhary et al., 2017).

## Applications of Bacteriogenic PtNPs

**FIGURE 4:**
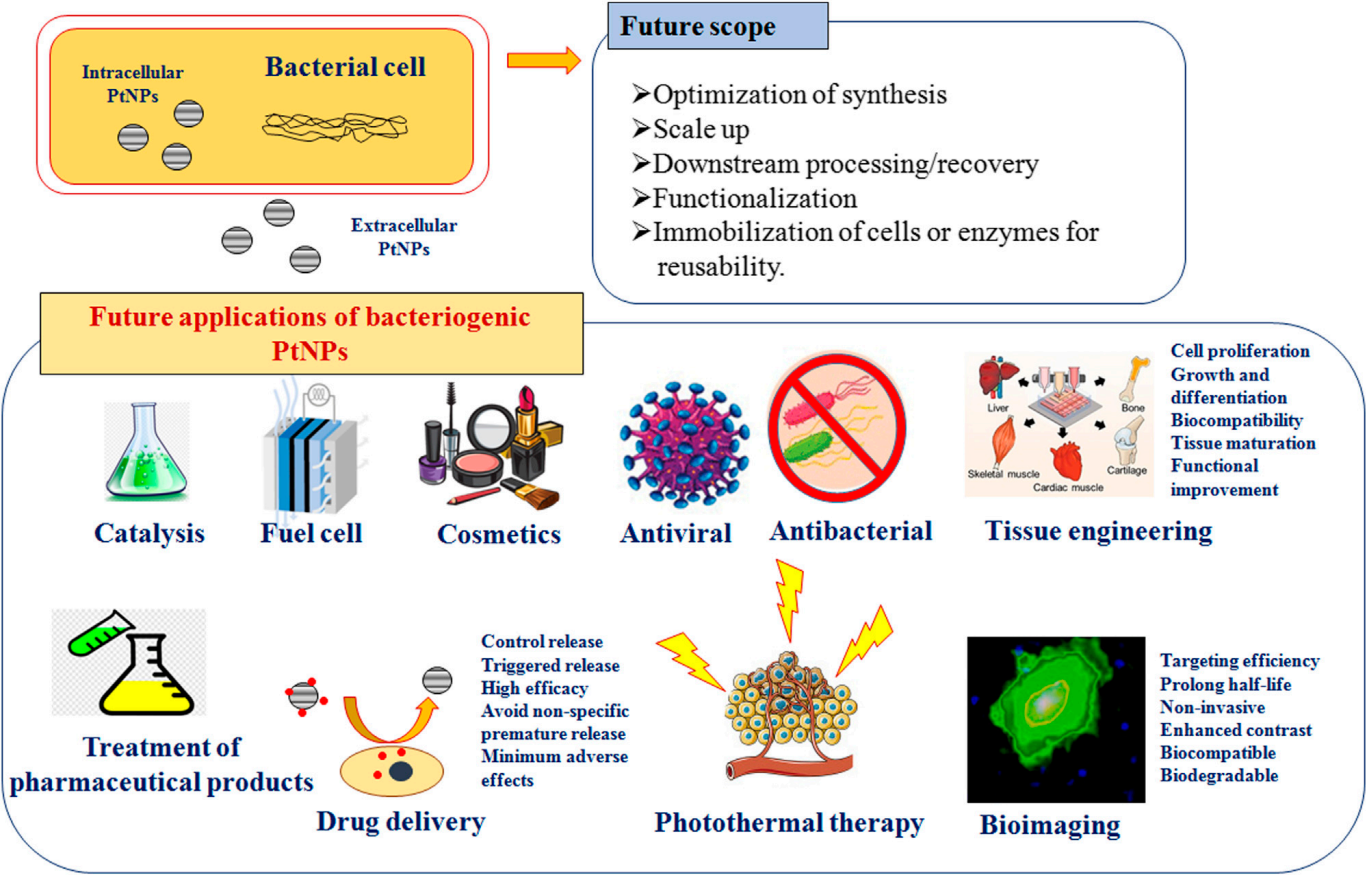
Scope and various applications of bacteriogenic PtNPs.

Several unique features of PtNPs such as surface functionalities, size distribution, shape, surface area, composition, crystalline nature, catalytic, thermal and plasmonic properties, have led to their application in various fields. PtNPs have also been used for several biomedical applications such as anticancer, antiinflammatory, nanodiagnostic, antibacterial, photothermal and radiotherapy, drug delivery and bioimaging (Figure 4). Several types of NPs are used for drug delivery, most notably being carrier for delivery of antibiotics. The small and controllable size of NPs shows antimicrobial operations and combat intracellular bacteria. Several metallic NPs like Pt, Ag, Au, Pd, ZnO and Cu show negative zeta potential and hence cell damaging potential. PtNPs exhibit more negative zeta potential and cause damage to cells hence enhancing antibacterial activity. However, bacteriogenic PtNPs are explored till date for limited applications which are discussed in the following section.

## Treatment of Pharmaceutical Products

The PtNPs synthesized from *D. vulgaris* (DSM 644) showed excellent catalytic activity in removal of pharmaceutical products (PhP). Four different classes of PhP (ibuprofen, ciprofloxacin, sulfamethoxazole and 17β-estradiol) are most relevant in the environment. PtNPs adsorbed 35% ciprofloxacin and 11% of both sulfamethoxazole and 17β-estradiol and acted as efficient biocatalyst in removal of PhPs. The efficiency of the adsorption was enhanced when the PtNPs catalysts were activated with hydrogen. After 24 h, 85% of sulfamethoxazole was removed at rate constant of 0.093 h^−1^ by PtNPs while 70% of ciprofloxacin and 94% of 17β-estradiol were removed with removal rate constants of 0.085 and 0.082 h^−1^, respectively. Toxicity of the molecule produced from the reaction of PhP with PtNPs was checked by estrogenic activity. Low level of estrogenic activity was found (71%) aiding in production of less toxic products. It is important to note that only 13% of the catalytic activity was lost during recycling, which indicates the reusability of bacteriogenic PtNPs for technological development towards treatment of pharmaceutical effluent (Martins et al., 2016).

## Anticancer Activity

The PtNPs synthesized from *Streptomyces* sp. exhibited promising anticancer activity against human breast cancer cell line (MCF-7). Treatment with PtNPs resulted in pronounced cellular deformity, detachment from the surface, and reduction in cell viability. In presence of bacteriogenic PtNPs only 52.77% cell viability was observed in MCF-7 human cell lines. IC_50_ value was found to be 31.2 µg/mL. Anticancer property of bacteriogenic PtNPs was found to be dose-dependent (Baskaran et al., 2017).

## Catalytic Activity

Catalytic reduction of 4-nitrophenol (4-NP) and azo dyes were carried out using Pd-Pt alloys NPs synthesized from *S. oneidensis* MR-1. The peak centered of 4-NP was found near about 318 nm and was shifted to 400 nm after addition of NaBH_4_. Pd-Pt alloys NPs were added which resulted in decrease in absorbance at 400 nm and increased at 300 nm indicating reduction of 4-NP to 4- aminophenol (4-AP). Within 4 min the reduction reaction was completed. It was concluded that Pd-Pt NPs located on surface of the biomass aided in transfer of electron from BH_4_^-^ ions to nitro group of 4- NP. Azo dyes such as methyl orange (MO), methyl red (MR) and acid red 14 (AR 14) was used for the reduction reaction. The absorption peak was found at 465, 431 and 506 nm for MO, MR and AR 14 respectively. Upon addition of Pt-Pd alloys NPs, the absorbance of azo dyes were decreased within 2, 6, 13 min in case of MO, MR, AR 14 respectively (Xu et al., 2019). The catalytic efficiency of PtNPs synthesized in presence of AQDS was enhanced and the K_app_ value was 0.0065 min^−1^ that might have attributed due to reduction in particle size (Tuo et al., 2016).

## Antibacterial and Antioxidant

PtNPs synthesized by microbial cell lysate supernatant of various bacteria showed both antioxidant and antibacterial efficiency. The antioxidant potential was checked by 2,2-diphenyl-1-picrylhydrazyl (DPPH) radical scavenging activity whereas the minimum inhibitory concentration (MIC) was used to check antimicrobial property. Purple color of DPPH was converted to pale yellow in the presence of PtNPs hence indicating that PtNPs participates in transferring of electron/hydrogen and neutralizes the DPPH radical. The antioxidant activity was found to be dose-dependent. At 1000 µg/mL concentration of PtNPs, various microbial strains showed different antioxidant activity. Highest antioxidant activity was observed in strain ZCI5 followed by FZC6, CCV1, NRRL B-11177, KC19, KT2440, ADR19 and at lowest MN23. DPPH free radical scavenging activity up to 95% was observed in ZC15 and FZC6 while 70% activity was seen in case of NRRL B-11177, KC19, KT2440 and CCV1 strains.

The antimicrobial activity was checked against *E .coli* and *S. aureus.* It was observed that, the surface electrical charge of NPs has significant impact in antibacterial activity. The zeta potential of synthezied PtNPs from Gram-negative organisms was found to be −36.88, −29.37 and −21.60 mV for strains named ADR19, FZC6 and NRRL B-11177, respectively and for Gram-positive it was −35.64, −30.90 and −26.08 mV for strains ZC15, KC19 and MN23 respectively. The zeta potential of genetically engineered bacterium was −20.37 mV. Although, PtNPs synthesized from NRRL B-11177 and KT2440 with similar zeta potential showed different activity against *S. aureus* and *E. coli*. This difference might be because of different size of NPs and peptidoglycan layer. The smaller size of PtNPs synthesized from NRRL B-1117 (3.84 nm) was easily able to pass through cell wall of *S. aureus* and cause reactive oxygen species (ROS) production, hence resulted in better antibacterial activity (Eramabadi et al., 2020).

## Scope and Future Applications

Although, physically and chemically synthesized PtNPs are used for various applications in catalysis and sensors, biologically synthesized PtNPs are yet to be explored for multivariate applications that are depicted in Figure 4. Hence, there is a need for critical consideration of using the greener path for synthesis of PtNPs. Bacteriogenic PtNPs can be used to develop amperometric gas sensor for hydrogen by electrodepositing on gold microchannel electrodes. Similarly, heavy metal ion sensors can be designed for environmental monitoring by depositing bacteriogenic PtNPs on tantalum substrate using ion beam sputtering deposition (IBSD) technique (Hussain et al. 2019). Implantable amperometric biosensors can be designed by immobilizing enzymes like glutamate oxidase on electrodes composed of bacteriogenic PtNPs, multi-walled carbon nanotubes and suitable conducting polymers. These types of sensors can be used for glutamate monitoring during traumatic injury (Nguyen et al. 2019). Further, bacteriogenic PtNPs should also be checked for catalytic combustion of hydrocarbon and oxygenated fuels for providing alternative power source for portable electronic devices. In this respect, it would be of utmost interest to evaluate the catalytic potential of these biogenic PtNPs to yield self-ignition and stable combustion from methanol-air mixtures (Guggilla et al. 2019).

Many bacteria synthesize PtNPs intracellularly or the particles are present in the periplasmic space of the bacterial cells. Similarly, in some cases the PtNPs are strongly bound to the cell surface. Hence, the recovery of the nanoparticles after synthesis is also another aspect which is needed to be addressed.

The rational optimization of the process parameters can overcome the aforementioned drawbacks. Firstly, the strain selection, which is most crucial for getting the PtNPs of desired dimension. Secondly, the cell passage and density and the reaction conditions (such as temperature, pH, aeration, agitation and concentration of metal salts), which should be optimized in order to ensure the reproducibility of the morphological features of bacteriogenic PtNPs. Various physical disruption methods such as grinding, mechanical disruption, freeze-thaw cycles, liquid homogenization, and sonication can be applied for recovery of the PtNPs by lysis of the cells. Similarly, chemical agents like organic solvents (e.g., alcohols, ether, and chloroform), chelating agents (EDTA), detergents or surfactants (e.g., SDS, Triton) and chaotropic agents (e.g., urea, guanidine) can be employed for bacterial cell lysis (Islam et al. 2017). Also the porosity of the bacterial cells can be increased by treating with calcium chloride so that the intracellular PtNPs can be tapped.

Another important aspect is application of genetic engineering and/or recombinant DNA technology to develop industrially important bacterial strains with high and efficient PtNPs producing ability. Such strain improvement method will increase the platinum metal salt tolerance of the bacteria, platinum ion adsorbing ability, and enhanced platinum ion reducing efficiency.

Although silver and gold nanoparticles from bacteria have been extensively studies for their therapeutic applications, only few studies have been reported for bacteriogenic PtNPs. Antibacterial, antifungal, antibiofilm, antiprotozoal, anticancer, antioxidant, anti-inflammatory and wound healing properties of bacteriogenic PtNPs should be explored. Similarly, thorough *in-vitro* and *in-vivo* toxicity studies must be performed to ensure that no adverse effects are caused during therapeutic applications. Thus, there is a huge scope of further research to explore bacteriogenic PtNPs and their applications.

## Conclusion

PtNPs have numerous applications in catalysis and fuel cell, while it has recently gained much attention for biomedical applications that include nanozymes, nanocarriers, nanosensors. Multifunctionalized PtNPs may prove to be efficient theranostic agent (with combined functionality for therapy and diagnosis). In this review we have compared the advantages and disadvantages of bacteriogenic synthesis of PtNPs with that of the other conventional physical and chemical methods. However, till date limited bacterial diversity has been exploited for the synthesis of PtNPs which is an environmentally benign rapid and efficient process. The enzymatic and non-enzymatic processes employed by bacterial cell for reduction of the Pt ions to PtNPs offer a better control over size distribution, shape and crystallinity. Furthermore, we discussed the toxicological effects, biomedical applications, and use of PtNPs in therapy and waste treatment. Although, bacterogenic PtNPs exhibit high efficacy with low concentrations, several factors still need to be considered before it can be used as a nanomedicine that include type of precursor, the method of production, stability, solubility, biodistribution, controlled release, bioaccumulation, cell-specific targeting, retention and toxicological issues to human beings. The future of bacteriogenic PtNPs in biomedical applications holds great promise, especially in the area of drug delivery, targeted therapy, early disease diagnosis, cellular and deep tissue imaging, gene delivery, as well as multifunctional therapeutics. In view of the background, it can be concluded that in future, multifunctional bacteriogenic PtNPs would be promising candidate for biomedical applications as novel nanomedicine.
